# Sarcopenia severity is related to arterial stiffness and hypertension in older Korean population without underweight and obesity: population based cross-sectional study

**DOI:** 10.3389/fpubh.2024.1469196

**Published:** 2024-10-30

**Authors:** Bokun Kim, Gwon-Min Kim, Up Huh, Juhyun Lee, Miju Bae

**Affiliations:** ^1^Future Convergence Research Institute, Changwon National University, Changwon, Republic of Korea; ^2^Human Community Renovation Research Center, R Professional University of Rehabilitation, Tsuchiura, Japan; ^3^Medical Research Institute, Pusan National University School of Medicine, Yangsan, Republic of Korea; ^4^Department of Thoracic and Cardiovascular Surgery, Pusan National University School of Medicine, Biomedical Research Institute, Pusan National University Hospital, Busan, Republic of Korea; ^5^Department of Thoracic and Cardiovascular Surgery, Samsung Medical Center, Seoul, Republic of Korea

**Keywords:** arterial stiffness, hypertension, obesity, older adults, sarcopenia, underweight

## Abstract

**Background:**

Aging and obesity are considered causes of arterial stiffness, which triggers hypertension in the older population. However, a substantial number of older adults without obesity have hypertension, suggesting that arterial stiffness and hypertension are related to different risk factors in older adults without obesity. This cross-sectional study aimed to determine whether sarcopenia is related to arterial stiffness or hypertension in older Korean adults without underweight and obesity.

**Methods:**

A total of 2,237 male and female adults in the Korea National Health and Nutritional Examination Survey who were ≥60 years and did not have underweight and obesity (18.5 ≤ body mass index <25.0 kg/m^2^) were involved. They were classified as moderate- (*n* = 276) or severe-sarcopenia (*n* = 528) as their sarcopenia index was 1 or 2 standard deviations lower than the mean of the young reference group. Arterial stiffness was confirmed using an estimated pulse wave velocity (ePWV) formula, and hypertension was diagnosed based on blood pressure or antihypertensive medication use.

**Results:**

Arterial stiffness and systolic and diastolic blood pressure showed an increasing trend from normal to moderate-to-severe sarcopenia (*p* < 0.001 for both). The distribution of subjects in the highest ePWV tertile and hypertension from normal to moderate-to-severe sarcopenia showed an increasing trend (*p* < 0.001 for both). Subjects with moderate or severe sarcopenia were 3.545 or 8.903 times more likely to be in the highest tertile of ePWV, and those with moderate or severe sarcopenia were 2.106 or 11.725 times more likely to be hypertension (*p* < 0.001 for all).

**Conclusion:**

Sarcopenia severity is related to arterial stiffness and hypertension in older Korean populations without underweight and obesity.

## 1 Introduction

Recently, South Korea has been considered the fastest aging nation in the world (1). As the older population rapidly increases, hypertensive heart disease is considered one of the most common causes of death in the older Korean population, which indicates that prevention and management of hypertension are critical (2). Many human and animal studies have suggested that arterial stiffness caused by aging and obesity is the triggering factor for hypertension onset in the older population (3–6). Interestingly, however, there are a substantial number of older Asian adults without underweight and obesity (18.5 ≤ body mass index < 25.0 kg/m^2^) who have hypertension (7). These findings show that arterial stiffness and hypertension progression are related to different risk factors in older adults without underweight and obesity.

Sarcopenia, which is characterized by an aging-related reduction in muscle mass, is a common geriatric syndrome and is considered a significant cause of metabolic impairment, physical disability, increased mortality and several unfavorable aging progresses. For arterial stiffness or hypertension, Campo et al. (8) reported that muscle mass rather than fat mass is linked to arterial health conditions and endothelial dysfunction, and Abbatecola et al. (9) reported that arterial stiffness is related to muscle mass reduction independent of body fat (8, 9). These studies indicate that sarcopenia may negatively affect arterial stiffness or hypertension, particularly in older adults without underweight and obesity.

The underlying mechanisms behind this unfavorable association include metabolic abnormalities such as increased oxidative stress, chronic inflammation, and insulin resistance, which are typically associated with aging and obesity (10–12). However, a significant number of older Asian adults exhibit metabolic abnormalities, even though they are not obese (13). This underscores the need to consider metabolic abnormalities as a critical factor in this population. Metabolic abnormalities not only drive muscle mass reduction but also serve as a significant risk factor for arterial stiffness and hypertension (11, 12), so sarcopenia may be closely related to arterial stiffness and hypertension in older adults without underweight and obesity.

Despite these potential connections, most of the previous studies that have investigated the associations between sarcopenia and arterial stiffness or hypertension have concentrated on individuals with obesity, leaving a gap in understanding for those who are neither underweight nor obese. Thus, this population-based cross-sectional study aimed to determine whether sarcopenia is related to arterial stiffness or hypertension in older Korean adults without underweight and obesity.

## 2 Materials and methods

### 2.1 Study design and subjects

**Figure 1:**
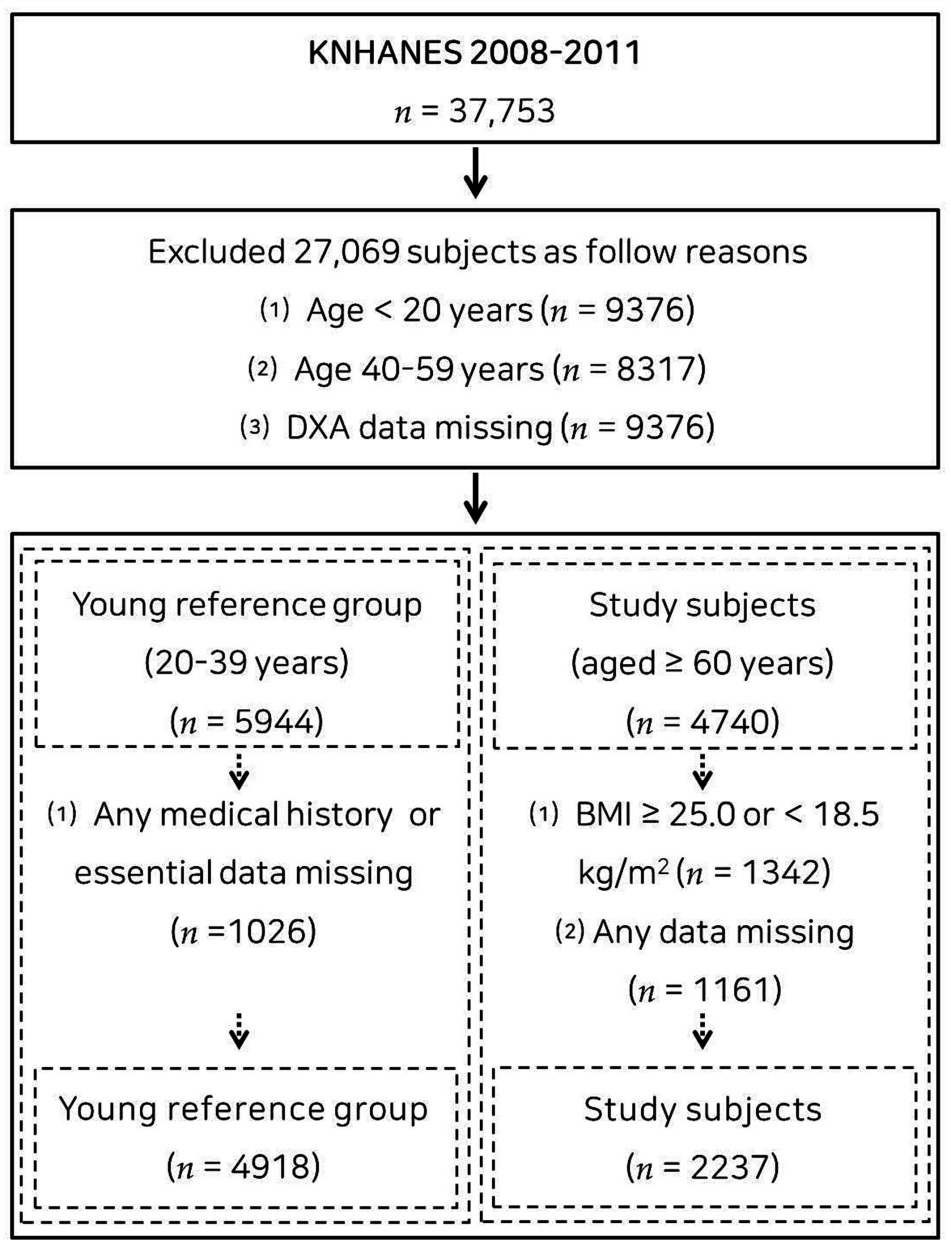
Flow chart of the study subjects.

This population-based cross-sectional study utilized data from the Korea National Health and Nutritional Examination Survey (KNHANES) of 2008–2011, an ongoing surveillance system in the Republic of Korea that assesses the general health, dietary intake, and lifestyle of the Korean population, as well as monitoring trends in health risk factors and the prevalence of primary health conditions. Of the 37,753 participants in the KNHANES 2008–2011, 4,918 adults aged 20–39 years were included in the young reference group (Supplementary Table S1), and 2,237 (1,113 males and 1,124 females) older adults without underweight and obesity (18.5 ≤ body mass index <25.0 kg/m^2^) aged ≥60 years were included in the study group (14). A flow diagram of participant recruitment is displayed in Figure 1. Each subject provided written informed consent. This study was carried out under the principles of the Declaration of Helsinki and approved by the Institutional Review Board of Changwon National University (7001066-202207-HR-051).

### 2.2 Anthropometric and sarcopenia evaluation

The subjects’ heights were assessed to the nearest 0.1 cm, and their body weights were assessed to the nearest 0.1 kg utilizing digital electronic scales. Body mass index (BMI) was computed as body weight (kg)/height (m)^2^. Obesity and underweight were defined on the basis of the 2022 update of the clinical practice guidelines for obesity by the Korean Society for the Study of Obesity, which confirms that a BMI of <18.5 or ≥25.0 kg/m^2^ is considered to indicate underweight or obesity, respectively (7).

Dual-energy x-ray absorptiometry (Discover-W fan-beam densitometer; Hologic, Marlborough, MA, United States) was used to assess the body composition of the subjects. For the sarcopenia index, appendicular skeletal muscle mass (ASM) was computed as the arm and leg lean mass minus the arm and leg bone mass (15–17). The values were subsequently divided by body weight and are expressed as a percentage ((ASM/body weight) × 100). Moderate and severe sarcopenia were indicated when the sarcopenia indices were one and two standard deviations (SDs), respectively, less than the sex-specific mean of the young reference group (n = 4,918) based on the methods of Janssen et al. (18). Many studies have adopted this muscle mass-focused method to define sarcopenia, and the reliability and validity were adequately verified (19–23). Since the means of sarcopenia index in young reference males and females were 33.10 ± 2.92 and 26.22 ± 2.47, respectively (Supplementary Table S1), the computed sex-specific cutoff points of moderate or severe sarcopenia were 30.18% or 27.26% for males and 23.75% or 21.28% for females, in the present study. Patients in the young reference group with a history of severe diseases, including cancer, stroke, other cardiovascular diseases, or arthritis, were excluded from averting any effects on the reference points. The characteristics of the young individuals in the reference group are shown in Supplementary Table S1.

### 2.3 Arterial stiffness and hypertension

Carotid-femoral pulse wave velocity (cfPWV) is the gold standard for assessing arterial stiffness. However, because measuring cfPWV requires advanced equipment and is challenging to implement widely, the need for a simpler, more accessible method has been recognized. The estimated pulse wave velocity (ePWV) equation provided by Greve et al. (24) offers such an alternative. The validity and reliability of the ePWV equation have been confirmed in many studies, including those focused on Korean individuals (25, 26). To estimate arterial stiffness, ePWV was computed as follows: ePWV = 9.587 – (0.402 × age) + (4.560 × 10^−3^ × age^2^) – (2.621 × 10^−5^ × age^2^ × mean arterial pressure (MAP)) + (3.176 × 10^−3^ × age × MAP) – (1.832 × 10^−2^ × MAP) (24). The MAP was computed as DBP + 0.4 (SBP-DBP) (24). Participants were classified into tertiles of ePWV to evaluate the severity of arterial stiffness.

The participants’ blood pressure was manually measured three times during the mobile health check-up, and the mean values were calculated. Hypertension was diagnosed when systolic blood pressure (SBP) was ≥130 mmHg, diastolic blood pressure (DBP) was ≥85 mmHg or medication was used to control blood pressure (27).

### 2.4 Other parameters and covariates

Parameters that are suspected or known to be related to sarcopenia, arterial stiffness and hypertension were included as covariates: sex, household income, educational level, medication use, alcohol consumption, smoking habit, moderate-to-vigorous physical activity (MVPA), dietary intake, WC and biochemical parameters (28–32). In the mobile examination center (MEC), face-to-face interviews examined household income, educational level, and medication use. Alcohol consumption, smoking habits, and MVPA were self-administered in the MEC. Dietary intake was examined via face-to-face interviews in participants’ homes, and other covariates, including WC and biochemical parameters, were measured and examined in MEC. Moreover, the quality of the laboratory data control program monitors laboratory performance to guarantee that all the analytical values meet acceptable standards of precision and accuracy. Household income was classified using tertiles. Alcohol consumption was categorized as never, ≤1 time/week, 2–3 times/week, or ≥4 times/week; education level as primary, middle and high school, or college or beyond; and smoking habits as never, former, or current smoker. Medication use was recorded depending on a self-reported diagnosis by a physician. MVPA was evaluated using the short form of the International Physical Activity Questionnaire. The reliability and validity of the questionnaire were verified in 12 nations, and the questionnaire has been adopted globally to evaluate physical activity in recent years (33). Total intake calorie data were gathered using a food frequency questionnaire, which was designed as an open-ended survey for reporting a variety of dishes and foods utilizing the 24-h recall method with various measuring aids. WC was assessed to the nearest 0.1 cm using a measuring tape. Blood samples were taken in the morning after a fast of >8 h. The circulating concentrations of glucose, hemoglobin A1c (HbA1C), total cholesterol, high-density lipoprotein-cholesterol (HDLC) and triglycerides were measured by enzymatic methods using a Hitachi automatic analyzer 7600 (Tokyo, Japan).

### 2.5 Statistical analysis

**Figure 2:**
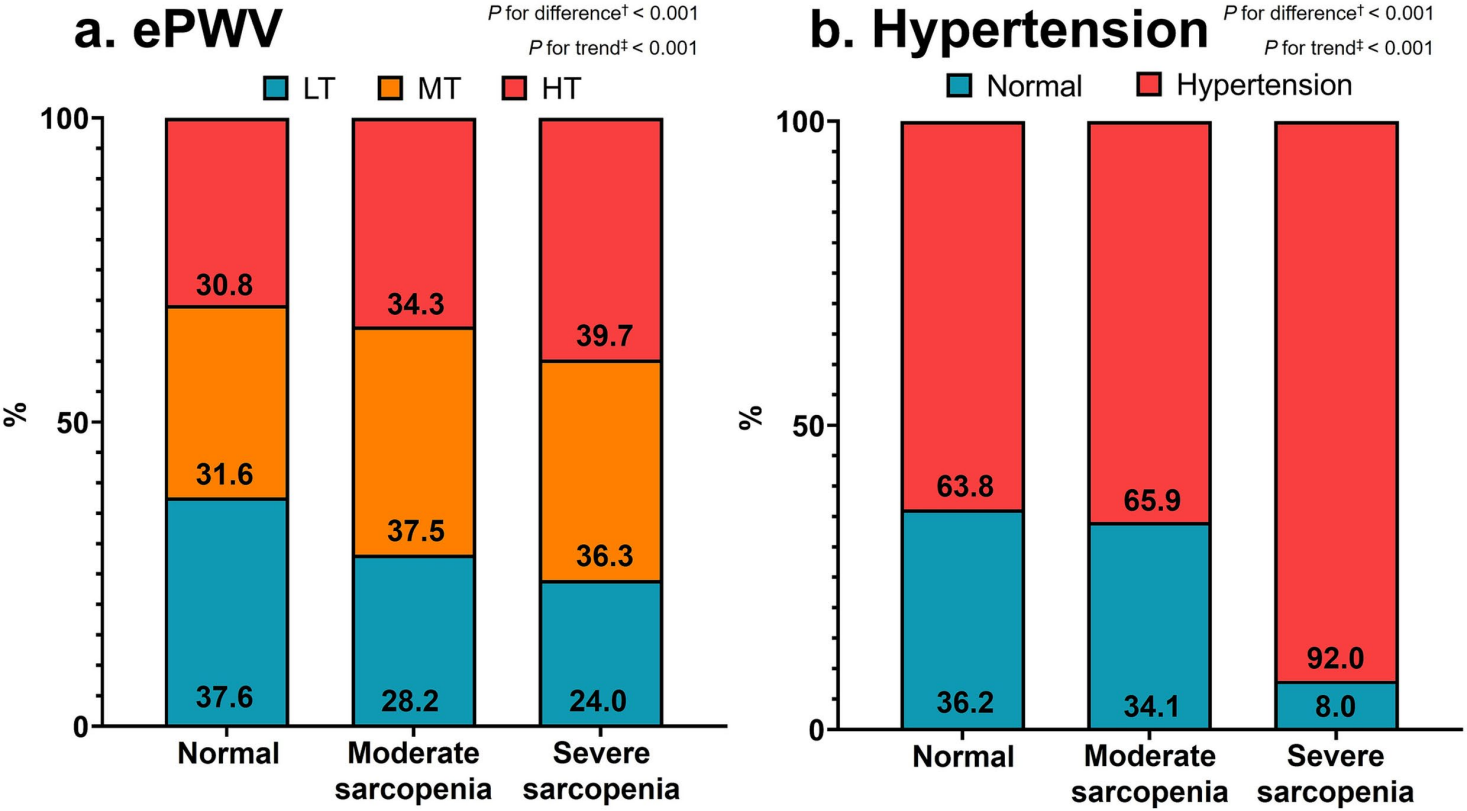
Distributions of ePWV tertiles (a) and hypertension (b) according to sarcopenia severity. Values are percentages of subjects. ^†^Chi-square tests and ^‡^linear-by-linear association methods were adopted for categorical differences and trends, respectively. ePWV, estimated pulse wave velocity; LT, lowest tertile; MT, middle tertile; HT, highest tertile.

Statistical analyses were conducted using SPSS software, version 26.0 (IBM Corp., Armonk, NY, United States). Except for age, all the data were weighted by age. The data are presented as the mean ± standard deviation (SD) or odds ratio (OR) and 95% confidence interval (CI). The independent-samples *t* test or the Mann–Whitney U test was adopted to compare parameters between men and women (Table 1). One-way ANOVA was employed to evaluate the characteristics and trends of the subjects according to sarcopenia severity (Table 2). Bonferroni correction was applied for pairwise comparisons when ANOVA indicated a significant difference, and the Mann–Whitney U test was employed for nonnormally distributed data verified based on Levene’s test for equality of variances. The Jonckheere–Terpstra test was adopted to determine trends in parameters among the three groups (two-tailed). The distributions of ePWV tertiles and hypertension according to sarcopenia status were compared utilizing chi-square tests, and linear-by-linear associations were adopted to reveal tendencies among all categories (Figure 2). Logistic regression was adopted to clarify the association between sarcopenia severity, which was an independent parameter, and arterial stiffness and hypertension, which were dependent parameters (Tables 3, 4). As mentioned above, the fully adjusted model was adjusted for the covariates of sex, household income, educational level, medication use such as antidyslipidemic drugs, alcohol consumption status, smoking status, moderate-to-vigorous physical activity (MVPA), total, intake calorie, WC and biochemical parameters (including HbA1c, glucose, total and HDL cholesterol, triglyceride). *p* < 0.05 was considered to indicate statistical significance.

**Table 1:** Characteristics of study subjects.

	Overall subjects (*n* = 2,237)			Male subjects (*n* = 1,113)			Female subjects (*n* = 1,124)			*p* value
Sarcopenia index^†^	28.51	±	4.11	28.48	±	4.21	28.54	±	4.01	<0.001
ePWV	11.01	±	1.26	10.91	±	1.25	11.11	±	1.26	<0.001
SBP, mm Hg^†^	126.48	±	15.14	123.39	±	11.49	129.54	±	17.51	<0.001
DBP, mm Hg^†^	76.00	±	9.10	75.52	±	8.61	76.48	±	9.54	<0.001
Hypertension medication use, %	43.9			42.3			45.5			<0.001
Age, year	68.9	±	5.8	69.0	±	5.7	68.8	±	5.8	=0.368
Height, cm^†^	157.10	±	8.73	157.89	±	8.79	156.31	±	8.59	<0.001
Body mass, kg^†^	54.49	±	6.98	55.51	±	7.06	53.47	±	6.75	<0.001
Body mass index, kg/m^2†^	22.03	±	1.68	22.22	±	1.67	21.84	±	1.66	<0.001
ASM, kg^†^	15.63	±	3.51	15.92	±	3.65	15.34	±	3.35	<0.001
MAP, mmHG^†^	96.19	±	10.12	94.67	±	8.54	97.70	±	11.27	<0.001

Values are means ± SD. ^†^Mann–Whitney U test was applied to assess the difference between groups. ePWV, estimated pulse wave velocity; ASM, appendicular skeletal muscle mass; DBP, diastolic blood pressure; MAP, mean arterial pressure; SBP, systolic blood pressure.

**Table 2:** Characteristics and trends of subjects according to the sarcopenia level.

	A. Normal (95% CI) (*n* = 1,433)				B. Moderate sarcopenia (95% CI) (*n* = 276)				C. Severe sarcopenia (95% CI) (*n* = 528)				*Post-hoc*	Trend^‡^
Sarcopenia index^†^	30.36	±	3.64	(30.33, 30.38)	26.73	±	3.01	(26.69, 26.77)	24.42	±	1.82	(24.40, 24.44)	A > B > C	<0.001
ePWV^†^	10.88	±	1.29	(10.87, 10.88)	11.14	±	1.15	(11.12, 11.15)	11.32	±	1.16	(11.30, 11.33)	A < B < C	<0.001
SBP, mm Hg^†^	123.72	±	17.47	(123.62, 123.83)	127.78	±	9.82	(127.64, 127.92)	133.29	±	4.90	(133.24, 133.34)	A < B < C	<0.001
DBP, mm Hg^†^	74.53	±	9.34	(74.47, 74.59)	78.07	±	7.72	(77.96, 78.18)	78.90	±	8.17	(78.82, 78.99)	A < B < C	<0.001
Age, year	68.9	±	5.8	(68.6, 69.2)	68.8	±	5.8	(68.2, 69.5)	68.8	±	5.6	(68.3, 69.2)	NS	=0.651
Height, cm^†^	159.14	±	8.86	(159.09, 159.20)	156.31	±	8.46	(156.19, 156.43)	151.94	±	5.85	(151.88, 152.00)	A > B > C	<0.001
Body mass, kg^†^	55.15	±	7.33	(55.10, 55.19)	54.52	±	7.38	(54.42, 54.63)	52.67	±	5.25	(52.62, 52.73)	A > B > C	<0.001
BMI, kg/m^2†^	21.71	±	1.66	(21.70, 21.72)	22.24	±	1.62	(22.22, 22.26)	22.79	±	1.49	(22.77, 22.80)	A < B < C	<0.001
ASM, kg^†^	16.84	±	3.50	(16.82, 16.86)	14.62	±	2.88	(14.58, 14.66)	12.86	±	1.61	(12.85, 12.88)	A > B > C	<0.001
MAP, mmHG^†^	94.21	±	11.25	(94.14, 94.28)	97.95	±	6.97	(97.85, 98.05)	100.66	±	5.65	(100.60, 100.72)	A < B < C	<0.001

Values are means ± SD. ^†^Mann–Whitney U test was applied to assess the difference between groups. ^‡^Jonckheere-Terpstra test was used to assess the trend among three groups. BMI, body mass index; DBP, diastolic blood pressure; ePWV, estimated pulse wave velocity; MAP, mean arterial pressure; NS, not significant; AST, aspartate transaminase; ALT, alanine aminotransferase; SBP, systolic blood pressure.

**Table 3:** Odds ratios for the relationship between sarcopenia level and ePWV.

	Unadjusted model		Fully adjusted model	
**The highest tertile of ePWV**				
Severe sarcopenia	2.021	(1.960–2.084)^***^	9.199	(8.452–10.012)^***^
Moderate sarcopenia	1.491	(1.434–1.551)^***^	3.400	(3.134–3.689)^***^
Normal	Reference		Reference	

Values are odds ratio (95% Confidence interval). ePWV = estimated pulse wave velocity. Fully adjusted model was adjusted for sex, house income, education level, hypertension and hyperlipidemia medication use, smoking, drinking, moderate to vigorous physical activity, nutrition, waist circumference, HbA1C, glucose, total and HDL cholesterol, triglyceride, aspartate transaminase and alanine aminotransferase.

***p < 0.001.

**Table 4:** Odds ratios for the relationship between sarcopenia level and hypertension.

	Unadjusted model		Fully adjusted model	
**Hypertension**				
Severe sarcopenia	6.527	(6.270–6.793)^***^	12.140	(11.184–13.178)^***^
Moderate sarcopenia	1.101	(1.065–1.137)^***^	2.168	(2.020–2.327)^***^
Normal	Reference		Reference	

Values are odds ratio (95% Confidence interval). Fully adjusted model was adjusted for sex, house income, education level, hyperlipidemia medication use, smoking, drinking, moderate to vigorous physical activity, nutrition, waist circumference, HbA1c, glucose, total and HDL cholesterol, triglyceride, aspartate transaminase and alanine aminotransferase.

***p < 0.001.

## 3 Results

Table 1 contains the study subjects’ characteristics. The mean sarcopenia index and ePWV of the subjects were 28.51 (SD, 4.11) and 11.01 (1.26), respectively, with those of males being significantly lower than those of females (*p* < 0.001). The mean SBP and DBP were 126.48 (15.14) and 76.00 (9.10), respectively, and the SBP and DBP in males were significantly lower than those in females (*p* < 0.001). Overall, 43.9% of the participants used hypertension medication, and 42.3 and 45.5% of the participants were males and females, respectively (*p* < 0.001). Supplementary Table S2 provides additional information on the subjects.

Table 2 displays the characteristics and trends of the subjects according to sarcopenia severity. A tendency test indicated a significant decrease in the sarcopenia index from normal to severe sarcopenia, but ePWV, SBP and DBP showed the opposite trend (all *p* < 0.001). Additionally, *post hoc* tests revealed significant differences among the three groups regarding the sarcopenia index, which decreased from the normal to severe sarcopenia groups. *Post hoc* tests revealed significant differences in ePWV, SBP and DBP among the three groups, which increased from the normal to severe sarcopenia groups. Supplementary Table S3 shows the results of the analyses of additional variables and several covariates in male and female participants.

Figure 2 displays the distributions of ePWV tertiles (a) and hypertension (b) according to sarcopenia severity, respectively. The distribution of ePWV tertiles significantly differed among the three groups (*p* < 0.001). However, the percentage of patients in the lowest tertile of ePWV decreased, but that of patients in the middle and highest tertiles of ePWV increased significantly from the normal to severe sarcopenia groups (*p* < 0.001 for both). The percentage of participants with hypertension significantly differed among the three groups and increased significantly from the normal to severe sarcopenia groups (*p* < 0.001 for both).

Table 3 compares the odds ratios for the relationship between sarcopenia severity and ePWV. For the highest tertile of the ePWV, according to the unadjusted model, the moderate and severe sarcopenia groups had odds ratios of 1.553 (95% confidence interval: 1.493–1.615) and 2.246 (2.176–2.317), respectively, compared with the normal group. According to the fully adjusted model, compared with those in the normal group, the odds ratios (ORs) in the moderate and severe sarcopenia groups were 3.545 (95% confidence interval: 3.266–3.848) and 8.903 (8.192–9.675), respectively.

Table 4 compares the odds ratios for the association between sarcopenia severity and hypertension. According to the unadjusted model, compared with the individuals in the normal group, the individuals in the moderate and severe sarcopenia groups had odds ratios of 1.101 (95% confidence interval: 1.065–1.137) and 6.527 (6.270–6.793), respectively. According to the fully adjusted model, compared with those in the normal group, the odds ratios (ORs) in the moderate and severe sarcopenia groups were 2.106 (95% confidence interval (CI): 1.964–2.258) and 11.725 (10.814–12.714), respectively.

## 4 Discussion

The purpose of the present population-based cross-sectional study was to explore the role of sarcopenia in arterial stiffness or hypertension in older Korean adults without underweight and obesity. The present study revealed that arterial stiffness and systolic and diastolic blood pressure significantly increased from normal to moderate-to-severe sarcopenia. The distribution of patients in the highest ePWV tertile and of hypertension from normal to moderate-to-severe sarcopenia showed a significant increasing trend. Subjects with moderate or severe sarcopenia were 3.545 and 8.903 and 2.106 and 11.725 times more likely to be in the highest tertile of ePWV and hypertension, respectively. Thus, sarcopenia severity is related to arterial stiffness and hypertension in older Korean population without underweight and obesity.

The results of the present study showed that sarcopenia severity is closely related to arterial stiffness and hypertension in older adults without underweight and obesity, which partly supports the findings of previous reports showing that the prevalence of these two conditions cannot be fully explained in older adults via obesity alone, even after adjusting for confounding factors such as lifestyle factors (34, 35). Additionally, previous studies, including Sanada et al. (36) and Han et al. (37), emphasized the close relationships of reduced muscle mass with arterial stiffness and hypertension. Sanada et al. (36) reported that subjects with severe sarcopenia had greater arterial stiffness than did those with normal or moderate sarcopenia, independent of sex. Han et al. (37) conducted an analysis that included the effects of obesity status and reported that sarcopenia plays a critical role in hypertension, independent of obesity. However, Kohara et al. (38) did not observe a close relationship between sarcopenia and arterial stiffness. This inconsistency may be derived from the definition of sarcopenia. Kohara et al. (38) defined sarcopenia as a sarcopenia index, which is calculated as body weight (kg) * skeletal muscle percentage, within the bottom 10% of all study subjects. However, Sanada et al. (36), Han et al. (37) and the authors of the present study utilized the sarcopenia index of young adults as a reference value to define normal, moderate or severe sarcopenia in study subjects. Because of the definition of sarcopenia, which is the aging-related reduction in muscle mass, it is essential to define whether older individuals have sarcopenia by comparing their data with those derived from the sarcopenia index of young adults. Considering all of these factors, it is commonly accepted that sarcopenia plays a critical role in arterial stiffness and hypertension, independent of obesity.

While sarcopenia is increasingly recognized as a critical factor, obesity and aging have long been established as primary contributors to arterial stiffness and hypertension. A study on young and middle-aged adults by Kim et al. (39) revealed that skeletal muscle mass index, percentage of fat mass and fat mass increased progressively across groups with higher body mass index (BMI): from adults without obesity (BMI < 25.0 kg/m^2^) to those with BMI ranges of 25.0–29.9 kg/m^2^, 30.0–34.9 kg/m^2^, and ≥35.0 kg/m^2^, but percentage of skeletal muscle mass decreased as BMI increase. This finding demonstrated that absolute values of skeletal muscle and fat mass and relative value of fat mass increase, but the relative value of skeletal muscle decreases as obesity rates increase (39). Since a combination of higher relative fat mass and lower relative muscle mass is associated with metabolic abnormalities such as oxidative stress, chronic inflammation, and insulin resistance, it negatively affects arterial stiffness and hypertension (23, 40). Conversely, young and middle-aged adults without obesity may be less prone to arterial stiffness and hypertension, compared to their counterparts with obesity.

However, it is not certain that all adults without obesity are free from the risk of arterial stiffness and hypertension. A study by Oshida et al. (41) utilized muscle ultrasonography to assess muscle quality in adults without underweight and obesity, focusing on the intramuscular fat in the rectus femoris and vastus intermedius muscles. The study revealed that age tended to increase in the modest, intermediate and poor muscle quality groups, whereas lean mass tended to decrease (41). Interestingly, the percentage of fat mass and fat mass did not significantly differ across the three muscle quality groups (41). Depending on these findings and implications, such as insulin resistance and inflammation, which are derived from poor muscle quality, aging-related adverse changes in skeletal muscle quantity and quality, rather than fat-related indices, may contribute to metabolic abnormalities in older adults without underweight and obesity (42, 43). However, the precise relationships among muscle quality, metabolic abnormalities, and their impact on arterial stiffness and hypertension remain unclear.

While the exact mechanisms are not fully understood, several potential explanations for the relationship between sarcopenia, arterial stiffness or hypertension have been proposed. Reduced muscle mass and intramuscular fat infiltration cause a decrease in insulin-responsive target tissue, resulting in insulin resistance; consequently, arterial stiffness increases, which indicates the onset of hypertension (44). The results of the present study supported this potential mechanism in that IR, evaluated using the triglyceride-glucose index, showed a significant increasing trend from normal to moderate-to-severe sarcopenia (Supplementary Table S2). Additionally, chronic inflammation may be a potential explanation for the relationships of sarcopenia with arterial stiffness and hypertension. This potential mechanism was also supported by the finding in the present study that WBC counts showed a significant increasing trend from normal to moderate-to-severe sarcopenia (Supplementary Table S2).

Furthermore, individuals with sarcopenia commonly exhibit functional impairment or physical disability, which induces a reduction in muscle contraction-derived anti-inflammatory markers called myokines (28). Since decreased myokine levels are an independent predictor of increased risk of sarcopenia and arterial issues, myokine deficiency in sarcopenia is more likely to increase the risk of arterial stiffness or hypertension (45–47). Unfortunately, the present study does not provide objective data on myokine deficiency in sarcopenia patients. However, given that sarcopenia is a common cause of functional impairment or physical disability, decreased myokine secretion in sarcopenia patients is reasonable. Finally, increased arterial stiffness may induce pulse pressure amplification in arteries. It may stimulate hypertrophy, remodeling or rarefaction in the microcirculation, which makes blood vessels unresponsive to the demand for changing blood flow, thereby leading to increased oxidative stress in muscles (48, 49). Oxidative stress damages muscle components, such as reducing the number and function of satellite cells, and may induce muscle mass reduction (50). Depending on all of these factors, sarcopenia may be a trigger of arterial stiffness or hypertension, and arterial stiffness or hypertension may worsen sarcopenia.

This study has both strengths and limitations. Significant potential covariates—including sex, house income, education level, medication use such as antidyslipidemic agents, smoking, drinking, moderate to vigorous physical activity, total intake calorie, waist circumference, HbA1C, glucose, total and HDL cholesterol, triglyceride—that may have a negative relationship with sarcopenia, arterial stiffness or hypertension were adjusted for in the present study. However, because of the cross-sectional nature of the study design, conclusions concerning the relationships of sarcopenia with arterial stiffness and hypertension in older Korean adults without obesity or underweight remain uncertain. Thus, longitudinal studies should be conducted to validate the conclusions of the present study. Additionally, since all the participants were older Korean adults, whether the results of the present study can be extrapolated to individuals of other ethnicities or in other nations is uncertain. Thus, additional investigations should be carried out in subjects of different ethnicities to assess the relationships of sarcopenia with arterial stiffness and hypertension in older adults without underweight and obesity.

In conclusion, as sarcopenia progresses in older adults without underweight and obesity, the relationship with arterial stiffness or hypertension becomes more apparent. These findings suggest that sarcopenia severity is related to arterial stiffness and hypertension in older Korean populations without underweight and obesity.

## Data Availability

Publicly available datasets were analyzed in this study. This data can be found here: The datasets generated during the current study are available in the 2008 to 2011 KHANES (https://knhanes.kdca.go.kr/knhanes/sub03/sub03_02_05.do) and analyzed during the current study are available from the corresponding author upon reasonable request.
